# Salvage Surgery for Severe Post-Bariatric Hypoglycemia After Multiple Bariatric Revisions: Reversing Roux-en-Y Gastric Bypass to Sleeve with Roux Limb as Henley-Longmire Interposition

**DOI:** 10.1007/s11695-023-06797-8

**Published:** 2023-08-30

**Authors:** Sebastian Holländer, Gereon Gäbelein, Ammar Al-Ali, Antonios Spiliotis, Philipp Robert Scherber, Matthias Glanemann

**Affiliations:** 1 Department of General Surgery, Vascular-, Visceral and Pediatric Surgery, Saarland University Medical Center, Kirrberger Str., 66421 Homburg, Germany; 2 Department of Surgery, Charité Universitätsmedizin Berlin, Charitépl. 1, 10117 Berlin, Germany

**Keywords:** Gastric bypass reversal, Dumping syndrome, Post-bariatric hypoglycemia, Complications after bariatric surgery, Roux-en-Y gastric bypass conversion to sleeve

## Introduction

Despite excellent weight loss and resolution of comorbidities experienced by most patients, Roux-en-Y gastric bypass (RYGB) is known to cause long-term complications that may necessitate revisional intervention or surgery. Post-bariatric hypoglycemia (PBH) is an increasingly recognized RYGB complication affecting 0.1–34% of patients [1–3], which can impact their quality of life [4]. In rare cases, reversing RYGB and restoring normal food passage may be considered due to severe PBH that is refractory to extensive therapeutic interventions [1, 5, 6]. However, reversal procedures carry a high risk of complications and usually require long operative times [7–10].

## Purpose

We demonstrate the feasibility of laparoscopic reversal of RYGB in a 38-year-old male patient who underwent multiple bariatric revision surgeries. The patient has a history of sleeve gastrectomy, conversion to RYGB, and subsequent procedures including two pouch resizings, hiatoplasty, and proximal vagotomy. The patient experienced severe, unresponsive PBH, with very low blood glucose levels (minimum: 18 mg/dL) concomitant with daily neuroglycopenic seizures.

## Methods

We provide an intraoperative video to demonstrate the laparoscopic restoration of normal food passage by utilizing the Roux limb as a Henley-Longmire interposition between the gastric pouch and remnant sleeve.

## Results

A side-to-side gastrojejunostomy between the remnant sleeve and the upper Roux limb was performed, avoiding dissection of the gastric pouch due to the risk of compromising its blood supply in the context of three previous surgeries. The remaining Roux limb was resected.

The operative time was 90 min. The postoperative course was uneventful. Upper GI contrast study on POD 2 showed no signs of leakage and a preserved tone of the pylorus. The patient was discharged on POD 4. At the short-term follow-up (3, 6, and 8 months post-surgery), the patient presented in good condition, reporting only 2–3 events per week with blood glucose levels between 50 and 60 mg/dl, accompanied by minor hypoglycemia symptoms. The lowest reported value was 45 mg/dl. No further seizures occurred.

## Discussion

Our understanding of the pathophysiological mechanisms underlying PBH is still incomplete [5]. Due to the rarity of RYGB reversal, the available literature on this topic is limited [6].

The clinical algorithm for diagnosing and treating PBH should be interdisciplinary and structured as a stepwise approach [5, 11], including dietary modifications and medication therapy if unsuccessful. In therapy-resistant situations, surgical or interventional revision of the gastroenterostomy, functional bipartition, or even reversal may be necessary. However, prior to the latter, temporary nutrition through a gastrostomy tube should be considered to assess the likelihood of success.

Although in our case, the patient’s symptoms improved and neuroglycopenic symptoms disappeared after the reversal of RYGB, it is important to note that restoring the normal food passage does not guarantee success. This highlights the incomplete understanding of the underlying pathophysiology and emphasizes the need for further basic research in this field.

## Conclusion

We believe that utilizing the Roux limb as an interposition is a safe and effective alternative to gastrogastrostomy, which we consider a high-risk anastomosis, especially after multiple prior revisions. This technique may be considered a salvage option in cases where conservative or interventional attempts have failed.

### Supplementary Information

Below is the link to the electronic supplementary material.

Supplementary file1 (MP4 231104 KB)
